# Food competition, but not other common assays, reveals social dominance in female and male CD1 mice

**DOI:** 10.21203/rs.3.rs-11078463/v1

**Published:** 2026-10-01

**Authors:** Elizabeth Illescas-Huerta, Hannah Casselman, Logan Taylor, Sequioa Smith, Nancy Padilla-Coreano

**Affiliations:** University of Florida; University of Florida; University of Florida; University of Florida; University of Florida

**Keywords:** Social dominance, social hierarchy, food competition, social behavior, female mice

## Abstract

Social dominance is fundamental to social organization across species and plays a critical role in shaping behavior, physiology, and health. Despite the complexity of social organization, researchers have successfully developed animal models to investigate social dominance under controlled experimental conditions across a variety of species, including male rodents. However, measures of social rank among female mice remain poorly understood, limiting the generalizability of commonly used social dominance assays. Here, we systematically evaluated four commonly used behavioral assays of social dominance in female CD1 mice and directly compared their performance with that observed in males. We found that three traditional assays—agonistic behavior, urine marking, and the tube test—failed to distinguish social rank in female CD1 mice. In contrast, the food competition task reliably identified social ranks in both female and male CD1 mice, revealing linear dominance hierarchies and demonstrating that this assay effectively assesses social dominance across sexes in an outbred mouse strain. By systematically comparing multiple behavioral assays in female and male CD1 mice, our study expands the current understanding of how social dominance is expressed across sexes and establishes a valuable platform for future studies aimed at uncovering the neural mechanisms underlying social hierarchy.

## Introduction

Social dominance hierarchies are conserved across many social species, whether they are in captivity or the wild^1,2^. Social hierarchies promote group stability and well-being, and help reduce conflict within social groups^3–6^. At the individual level, social rank in rodents is associated with differences in neural plasticity, stress, and immune responses^7–10^. Given these broad physiological and behavioral consequences, reliable methods for determining social rank are important across many areas of biomedical research. Dominance behavior in mice has been measured and studied in both wild^11,12^ and laboratory settings^13,14^. Dominance in mice often manifests through agonistic behaviors and resource competition, with higher-ranking mice expressing more agonistic behaviors and having a competitive advantage in food and territory resources^6,15–18^.

Several laboratory behavioral assays have been implemented to assess social ranks. Common approaches include the tube test^19–21^, observation of agonistic behaviors^9,14,16,22^, urine marking as a measure of territorial dominance^13,23,24^, and food competition tasks^14,17,25^. Food competition tasks have been shown previously to be effective in establishing dominance for a range of species, including mice^26^, voles^27^, and rats^28,29^. However, previous research on social dominance in rodent species has focused primarily on assessing social rank in males^6^. In mammalian social species, including rodents, females in the wild also compete for resources such as food and territory^30^ implying that dominance has an important social role across sexes. Despite the importance of resource competition in natural social interactions, food competition has not been well characterized as a measure of social dominance in laboratory female mice. Similarly, although wild female mice engage in territorial urine marking^23^, this behavior has generally been interpreted primarily in the context of reproductive communication^6,23^.

Recent work demonstrated that inbred C57BL/6 female mice exhibit robust urine scent marking independent of sexual motivation or social partner identity only when exposed to novel urine odors during adolescence, indicating that this behavior functions as a general form of social communication, rather than exclusive to mate selection^31^. Whether urine scent marking reliably reflects social dominance in female mice remains unknown. This demonstrates a significant gap in sex-specific understanding of dominance behaviors.

Importantly, sex differences have been reported in both the mechanisms underlying social dominance and the behavioral expression of dominance in rodents^32–35^. Female mice exhibit lower levels of agonistic behavior than males under some laboratory conditions^34^, raising the possibility that assays developed or validated primarily in males may not identify dominance relationships equally well in females. In this study, we directly compared commonly used measures of social dominance—agonistic behavior, tube test, urine marking, and food competition—and assessed their efficacy in identifying social rank to determine whether these measures differ between female and male mice. We further tested whether food competition reveals similarly structured dominance relationships across sexes. Because our previous work demonstrated that outbred CD1 male mice exhibit greater territorial marking and agonistic behavior than C57BL/6 male mice^14^, we used outbred CD1 mice to maximize the expression and detection of dominance-related behaviors.

## Results

### Female CD1 mice engage in fewer agonistic behaviors and allogrooming compared to male CD1 Mice

We first assessed dominance based on agonistic behavior in home cages with four resident mice. For both male and female CD1 mice, we observed interactions between cage mates in the home cage for 15 minutes per day over five days. We quantified who initiated and received fights, chases, and allogrooming behaviors (Fig. 1a). As reported in previous literature^16^, male-dominant mice display agonistic behavior toward subordinate cagemates. During our 15-minute daily observation period, males initiated fights and chases, whereas females did not (Fighting: t(28.0) = 3.506, p = 0.0016; chasing: t(28.0) = 2.048, p = 0.0500), suggesting that agonistic behaviors commonly seen in male mice and that are effective for measuring dominance may not be as useful in females. Since female mice are overall less involved in aggressive behavior^6^, we measured whether affiliative behavior could determine social dominance. Although females exhibited some allogrooming, males showed significantly more allogrooming behavior (Fig. 1b; t(36.52) = 3.329, p = 0.0020). Thus, our results show that females rarely engage in either agonistic or affiliative behaviors with cagemates in regular housing, suggesting sex-dependent differences in social interactions in CD1 mice. Therefore, under laboratory cage conditions, short daily observations of agonistic and affiliative behavior are ineffective for establishing ranks in CD1 female mice.

### Urine marking assays are ineffective to establish social dominance in female CD1 mice

Since territorial marking is common in rodents and an effective measure of dominance for male mice^23,36^, we next investigated whether territorial marking can establish dominance in female CD1 mice. In a pairwise manner, two cage mates were placed in a novel arena separated by a mesh wall (Fig. 1c), and the number of urine markings made by each mouse was quantified^14^. We measured the number of urine marks produced by paired mice to determine dominance relationships between each pair. The mouse with more urine marks was classified as dominant, whereas the mouse with fewer urine marks was classified as subordinate, as previously described^14^. We found that male mice showed significantly more urine spots than female mice across all the pair combinations tested (Fig. 1d, t(27.07) = 5.028, p < 0.0001). Moreover, the total proportions of male mice that showed dominant and subordinate ranks based on their urine spots were similar (Fig. 1d). Very few male mouse pairs were tied for the same dominance placement in the urine marking test. In contrast, most female pairs tied for the number of urine marks during the test, producing a larger proportion of mice that could not be identified as either dominant or subordinate. Our results suggest that although the urine marking test is effective for establishing dominance in CD1 males, it fails to adequately measure social ranks in female mice, indicating that CD1 female mice do not engage in marking behavior in the presence of cage mates.

### Female and male CD1 mice do not exhibit dominance behavior during tube testing (dup: abstract ?)

We then analyzed social dominance using the tube test, a common assay used for measuring social rank in laboratory mice^7,13,19,37^. We previously showed that CD1 males that are dominant in agonistic and territorial ranking are more likely to be losers in the tube test^14^. However, because the tube test is widely used in behavioral neuroscience and its application in female CD1 mice has been relatively understudied, we next examined whether this assay reliably identified social dominance in female CD1 mice, using males as a comparison group. After a minimum cohabitation period of two weeks, cagemates were first trained to walk through the tube alone to acclimate. We then tube-tested mice in a round-robin manner for seven days to stabilize outcomes. During the seventh test, we quantified four separate behaviors during trials for both winners and losers: push, resist, retreat in contact, and passive retreat (Fig. 2a). To determine whether females and males differed in their behavior during the tube test, we first compared trial duration. CD1 females had shorter tube trial lengths (Fig. 2b; t(45.83) = 3.754, p = 0.0005). However, tube test winners and losers showed no differences in pushing, resisting, or retreating times in female mice (Fig. 2c–d). Interestingly, there was a significant interaction of sex and outcome (winning or losing) for passive retreat behavior, showing that CD1 male losers retreated passively more than females, as well as significantly more retreat in loser than winner male mice (Fig. 2d; two-way ANOVA, sex × outcome F(1, 139) = 7.336, p = 0.0076; Post hoc comparison, Male: winner vs. loser trials, p = 0.0017; Loser trials: female versus male, p = 0.0206). Additionally, we found a significant main effect of sex on resistance behavior, with female CD1 mice exhibiting greater resistance than male CD1 mice, regardless of the trial outcome (Fig. 2c left; two-way ANOVA sex, F(1, 139) = 12.21, p = 0.0006), but with no significant differences between winner and loser across sex. Overall, our results suggest that the tube test does not capture interpretable dominance relationships in either sex of CD1 mice.

### Food competition test reveals dominance in female and male CD1 mice

Given that common dominance assays of agonistic behavior, territorial behavior, and the tube test did not yield dominance relationships in CD1 female mice, we developed a food competition assay. Competition for resources is another means of measuring dominance in male rodents, especially when animals are food-restricted and motivated^26,28,38^. Male and female food-restricted mice were individually trained for three consecutive days to pick up a small sugar pellet from a small cup feeder in an open arena with a gate on the opposite end (Fig. 3a, left). After three days of training, all cagemates learned to pick up the desirable sugar pellet within 120 seconds. Importantly, once fully trained, all mice always ran towards the feeder after the gate was lifted (Supp Videos 1–2). We performed round-robin pairwise competitions over two days, and recorded latencies to arrive at the feeder and eat the reward. In each trial, the mouse that picked up the reward first was considered the winner, while the other was considered the loser. Mice were ranked based on the total number of trials won, with ranks 1 and 2 classified as dominant and ranks 3 and 4 as subordinate.

We found that dominant females had a significantly shorter latency time than subordinates during competition (Fig. 3b; t(22) = 2.086, p = 0.0488), while males showed the same pattern (Fig. 3b; t(16) = 2.887, p = 0.0107). These differences between dominant and subordinate mice in both females and males were not attributable to differences in training or motivation to compete for the reward, as both groups exhibited similar latencies during the training (Fig. 3a; female, t(22) = 0.1617, p = 0.8730; male, t(16) = 0.2323, p = 0.8192), and similar body weight during competition (data not shown, Female, dom: 25.97 g, sub: 25.61 g; *t*(22) = 0.4438, p = 0.6615; Male, dom: 41.92 g, sub: 40.47 g, t(22) = 0.7174, p = 0.4835). To assess whether training performance predicted winner or loser outcomes during competition, we correlated training latencies with competition test latencies in both female and male CD1 mice. We found no significant correlations in either females or males, indicating that latency during the competition was influenced more by the presence of a competitor than by the animals' baseline performance during training (Fig. 3c–d). Additionally, we compared latencies during the food-competition task across social ranks (Rank 1–Rank 4). Female mice displayed significantly shorter latencies in Rank 1 than in Rank 4 (one-way ANOVA; F(3,20) = 3.212, p = 0.045; post hoc comparison, Rank 1 vs. Rank 4, p = 0.043), whereas males showed a similar, though non-significant, trend (one-way ANOVA, F(3,14) = 2.248, p = 0.1278; Fig. 3e).

Next, we examined whether females showed similar performance across the social hierarchy during the food competition test. (Fig. 3f). We found no significant difference between females and males in dominant versus subordinate latencies during competition, indicating that the relationship between social rank and competition latency was similar across sexes (two-way ANOVA; outcome × sex, F (1,38) = 2.451, p = 0.1257; outcome, F(1,38) = 0.2847, p = 0.5968; sex, F(1,38) = 0.6705, p = 0.4180) Finally, our food competition assay revealed that both female and male groups exhibited comparable Directional Consistency Index (DCI) and Landau's h values, indicating similarly linear and consistent dominance hierarchies across sexes (Fig. 3g; female, (t(9)= −0.055, p = 0.958); male, (t(9) = −0.785, p = 0.460). Taken together, our results showed that the food competition test successfully established a rank for both sexes and can be used to determine relative social rank in a pairwise manner.

## Discussion

Here, we evaluated four commonly used assays of social dominance in female and male CD1 mice. Our results demonstrate that the utility of the most common social dominance assays is sex-dependent in mice. Agonistic behavior and urine marking reliably identified social rank in males but not in females, whereas the tube test failed to distinguish social rank in both sexes. In contrast, the food competition assay consistently identified stable, linear social hierarchies in both female and male CD1 mice, establishing it as a robust measure of social dominance across sexes in this outbred strain.

### Overt agonistic and affiliative behaviors are not reliable indicators of social dominance in female CD1 mice

Agonistic behavior has long been considered a hallmark of social dominance in rodents, especially in males^6,39^. Although agonistic behaviors have been reported in female rodents^22,40–42^ we did not observe clear agonistic interactions among our female CD1 mice during standard laboratory homecage observations. The absence of overt agonistic behaviors in female mice may be attributable to stable social ranks at the time of observation. A previous study has shown that CD1 female mice more rapidly establish stable social hierarchies, often within the first few days of co-housing in a large enriched cage^22^. Our homecage observations occurred after several weeks of co-housing, suggesting that a dominance relationship may already have been established, reducing the need for overt agonistic interactions. In contrast, according to the literature^14,16^, CD1 male mice exhibited higher levels of agonistic behaviors, indicating fundamental sex differences in the expression and maintenance of social dominance. Interestingly, CD1 males also showed significantly more allogrooming behavior than females. Although allogrooming has primarily been investigated in a paradigm involving distressed conspecifics in both female and male mice^43^, spontaneous allogrooming during home-cage interactions between CD1 mice has received little attention. Based on previous literature on allogrooming as a prosocial behavior, this effect may be explored as a future direction for evaluating consolation behavior towards cagemates receiving agonistic behavior from dominant mice.

Future studies examining agonistic behavior during both the establishment and maintenance of social hierarchies in female mice are needed to better understand how dominance relationships evolve over time. Furthermore, complementary assays that more directly elicit agonistic interactions, such as the resident–intruder paradigm^42^, or in sexually experienced females, may provide additional insight into the mechanisms underlying female agonistic dominance, as previously reported in wild house female mice^44^.

### Territorial marking does not reliably reflect social dominance in female CD1 mice

Similar to agonistic behaviors, the urine marking assay is frequently used to measure social dominance in rodents, especially in males^6^. To our knowledge, no previous study has evaluated urine marking as a measure of social dominance in female laboratory mice. Although female urine marking has traditionally been associated with reproductive communication^44,45^, recent evidence indicates that laboratory inbred female mice can use urine scent marking as a general form of social communication, independent of sexual motivation or social partner identity^31^. Nevertheless, under our experimental conditions, female CD1 mice left few urine marks, preventing reliable discrimination of social rank. In contrast, male CD1 mice exhibited robust urine marking with clear differences between dominant and subordinate animals, supporting its utility as a measure of social dominance in males, consistent with previous studies in CD1 mice^14^. One possible explanation for these discrepancies is that previous studies were conducted in wild house mice or inbred laboratory mice and primarily examined urine marking as a form of social communication rather than as an indicator of social dominance. In contrast, our study assessed whether urine marking could reliably discriminate hierarchical status in outbred CD1 mice using a conventional urine-marking assay. Domestication, strain differences between outbred CD1 and inbred laboratory mice, or the limited sensitivity of conventional urine-marking assays may have contributed to our inability to detect dominance-related differences in female urine marking. Future studies using more sensitive approaches to quantify urine scent marking in outbred CD1 mice will be necessary to determine whether these findings reflect methodological limitations or genuine strain-specific differences.

### Female and male CD1 mice do not exhibit dominance behavior during tube testing

The tube test is one of the most widely used assays for assessing social dominance in rodents because it provides a simple and reproducible measure of competitive interactions^6,39,46^. Although the tube test has been widely used to assess social rank in both female and male mice, most studies have used inbred strains^19,20,47–50^. In contrast, relatively few studies have evaluated this assay in outbred male mice^14,51–53^, and none in female mice. Here, we found that the tube test was ineffective in determining social dominance in female CD1 mice, extending our previous findings in male CD1 mice^14^ and confirming that this assay is ineffective in both sexes. Notably, we found interesting sex differences in tube test performance. Females spent less time in the tube test than males, but exhibited greater resistance during direct competition and were less likely to retreat passively. These findings indicate that female and male CD1 mice exhibit different behavioral patterns during the tube test. However, because none of the behaviors distinguished winners from losers, this task was not useful for studying CD1 dominance behaviors.

### Food competition test shows dominance behaviors in CD1 female and male mice

Competing for limited resources is one of the most widely used approaches for analyzing hierarchical relationships in rodents^5,6,39^. Within this framework, food competition has been successfully used to determine social dominance in C57 inbred females^54,55^, but outbred females have not been studied in the lab setting. Here, we extend these findings by demonstrating that the food competition assay successfully identifies dominant and subordinate individuals in both female and male CD1 mice. Previous studies have assessed social dominance during food competition using several behavioral measures, including the total number of sucrose pellets obtained by each animal^28,38^, time spent eating^56^, the amount of food consumed^57^, and the order of access to the food resource^27,58,59^. Here, we used the latency to obtain food as a quantitative measure of social rank because it allowed us to directly compare individual performance during training and competition, thereby distinguishing differences in social rank from differences in task acquisition. Differences in latencies observed only during competition allowed us to classify animals by relative social rank across sexes and across all ranks for females, but that trend was not significant for males. One possible explanation is that female social hierarchies are expressed more consistently through priority access to food resources, whereas in males, additional behavioral factors may contribute to competitive performance, resulting in greater overlap between social ranks.

Notably, the hierarchies identified by food competition were highly linear and directionally consistent in both female and male CD1 mice, two defining characteristics of well-defined dominance hierarchies^5^. High directional consistency indicates that competitive interactions occurred in a predictable direction, with the same individuals consistently prevailing over their cage mates, whereas linearity demonstrates that individuals can be reliably ordered from dominant to subordinate within the social group^60,61^. This finding is particularly important because previous studies have suggested that female rodents exhibit lower hierarchy linearity in agonistic dominance than males^22^. In contrast, food competition identified highly linear and directionally consistent hierarchies in female CD1 mice, comparable to those observed in males. Together, these characteristics establish predictable social relationships. Such stable dominance hierarchies have been proposed to reduce repeated conflicts over access to resources^5^ and further support the validity of food competitions as a robust measure of social organization in both female and male CD1 mice. Moreover, this assay's ability to reliably assign social rank makes it well suited for studies investigating the behavioral and neural mechanisms underlying social dominance across sexes in outbred mice.

Our findings raise an important question about why food competition successfully identifies social rank in female CD1 mice, whereas other commonly used dominance assays do not. Although few previous studies have shown that agonistic behavior, urine marking, and the tube test can successfully assess social dominance in female mice^9,22,23^, we found that these assays failed to distinguish dominant from subordinate female CD1 mice, whereas food competition reliably identified social rank. One possibility is that female social hierarchies are expressed more consistently through priority access to food resources, whereas other behaviors, such as agonistic interactions or territorial marking, play a less prominent role in maintaining dominance relationships. Previous studies have suggested that not all species rely on aggression or territorial signaling to establish and maintain social hierarchies^5^. Similarly, our findings suggest that female and male CD1 mice may also differ in the behavioral strategies used to express social dominance.

### Limitations of the study and future directions

Several limitations of this study should be acknowledged. First, our findings were obtained exclusively in CD1 mice; therefore, it remains to be determined whether the effectiveness of food competitions generalizes to other mouse strains. Second, although female mice were tested without controlling for the estrous cycle, previous studies have suggested that social hierarchy is relatively stable across estrous stages^22^. Nevertheless, future studies could directly evaluate whether fluctuations in ovarian hormones influence performance in the food competition assay or the expression of social rank.

## Conclusion

Our findings demonstrate that the food competition assay, but not the other commonly used dominance assays evaluated here, effectively measured social dominance in both female and male CD1 mice, supporting its use for evaluating social hierarchies across sexes in CD1 mice. By providing a robust method to assess social hierarchy across sexes, this work establishes a valuable platform for future studies aimed at uncovering the neural mechanisms that underlie social dominance and its impact on brain function and behavior.

## Methods

### Animals

All experimental animals were maintained on a 12 h reverse light cycle (lights off: 8:30 A.M.–8:30 P.M.), and all behavioral testing was conducted during the dark phase between 9:00 A.M. and 6:00 P.M. Male and female CD1 mice were 8 weeks old upon arrival and group-housed (3 or 4 per cage). Behavioral experiments began at least one week after arrival, and mice were handled for at least 2 days before testing. Food and water were provided *ad libitum*, except during the food competition assay, in which animals were food-restricted to 90% of their baseline body weight. Mice were allowed to habituate to the dimly lit, quiet testing room for 15 minutes prior to any experiment. All procedures were performed in accordance with the University of Florida regulations and were approved by the Institutional Animal Care and Use Committee (IACUC).

### Behavioral Assays

#### Home cage observations

Observations of female (n = 24) and male (n = 29) CD1 mice were performed in a dimly lit room (20 lux), with water and food containers removed for clear observation. Trained observers watched all four mice interact for 15 minutes over five days. Three different behaviors were observed: fighting, chasing, and allogrooming. Fighting involved one mouse initiating aggressive behaviors, chasing involved one mouse running after another, and allogrooming was defined as one mouse grooming another mouse. The frequency of the behaviors, as well as which mouse was the initiator and which was the receiver, was recorded. The mouse that initiated the behavior was deemed dominant, with the receiver being the subordinate mouse.

#### Urine marking test

All testing from female (n = 24) and male (n = 29) CD1 mice was performed in a dimly lit room. The apparatus consisted of a clear plastic enclosure with no floor. A perforated clear wall split the enclosure in half, separating mouse pairs and preventing physical interaction. We placed chromatography paper on the enclosure floor and covered it with a wire mesh to prevent mice from eating it during testing. We placed mouse pairs from the same cage in the enclosure and allowed them to urinate for 2.5 hours. Urine marks were counted by hand under a UV light. For each pairwise comparison, the mouse with the greater number of urine marks was designated the winner, whereas the mouse with fewer urine marks was designated the loser. After completing all pairwise comparisons within a cage, we combined the outcomes to generate a cage-wide hierarchy by ranking mice by total pairwise wins. Each subject was tested once per day with a different cage mate in each session, so every mouse encountered each cagemate once during the assay.

#### Tube test

Tube test data from 24 male CD1 mice used in this study were previously reported in^14^; here, they were reanalyzed and compared with female CD1 mice (n = 24) tested in the same conditions for the present study. We conducted all training and testing in a dimly lit, quiet room. We used a narrow, restrictive tube that allowed only one mouse at a time to occupy the space for both training and testing. The tube used for both female and male mice was 30.48 long and made of clear polyvinyl chloride pipe. The inner diameter of the tube was 3.175 cm for CD1 female mice and 3.5 cm for male CD1 mice. The tube and surrounding area were cleaned using a paper towel between subjects and thoroughly sanitized with 70% ethanol between cages. A small plastic tube slightly smaller than the tube was used to gently prompt the mouse through the tube if it showed prolonged reluctance.

Training: Mice were trained for two consecutive days prior to testing. Training involved placing one mouse at the entrance of the tube by its tail and releasing the animal into the tube. The mouse was allowed to walk through the tube then prompted to turn around and re-enter the tube. This was performed until the mouse had walked through the tube ten times. This training protocol was done for each mouse in each cage.

Test: For each cage, mice were tested daily in a round-robin design against all other cagemates, with the order of pairings randomized across rounds. The starting side of the tube for each mouse alternated across testing days. Animals were picked up by their tails and released into the tube. The winner of the trial was defined as the mouse that forced the other mouse out of the tube, and the loser as the mouse that exited the tube with all four paws. Mice with more trial wins were considered more dominant, and mice with more trial losses were considered subordinate. Both female and male mice were tested for eight days, at which point the male mice showed a stable hierarchy. On the last day of tube testing, we measured four behaviors: pushing, resisting, active retreat, and passive retreat. Pushing was defined as the mouse moving or attempting to move forward and engaging in physical contact with the other mouse. Resisting involved one animal refusing to move and not engaging in physical pushing. Active retreat was defined as one animal retreating while still touching the other animal, while passive retreat involved one animal retreating without physical contact.

#### Food competition test

All training and testing of female (n = 24) and male (n = 18) CD1 mice were conducted in a dimly lit, quiet room. The food competition apparatus was a black-box arena with a manually operated gate. A small container in the upper corner contained one food pellet (20mg sucrose pellets, Bio-Serv). The arena was thoroughly cleaned with 70% ethanol between cages. Both female and male mice received 3 g of sucrose pellets in their home cages to reduce neophobia and were habituated to the arena for 10 minutes one day before training began.

Training: Mice were trained for three consecutive days. Three pellets were placed in a container in the upper corner of the arena. The container was counterbalanced between trials and days. One at a time, the mice were placed in the arena and given up to 10 minutes to eat the pellets, with most mice consuming between 1 and 5 pellets. After eating the pellet, the mice were left in the box for an additional minute to adjust to the environment. We recorded the time it took mice to consume the pellets. The third day of training involved adding a manually operated gate and only one sugar pellet in the container. Each mouse was placed behind the gate for 30 seconds, then the gate was lifted, and the mouse was allowed to consume the pellet. The latency time from the release from the gate to pellet consumption was recorded and used for the analysis.

Test: Three days of testing followed the training period. Mouse dyads were allowed to compete for one food pellet. Each mouse was exposed to each of their cagemates one at a time, resulting in six combinations. Each mouse participated in three trials per day, with 18 trials overall per cage each day. The mouse matches were randomized across trials, and the side of the container with the pellet was counterbalanced between pairs. The winner mouse was defined as the mouse that consumed the pellet first. We defined latency time as the amount of time between release from the gate and pellet consumption. We recorded latency time for trial one on the last day, and used it for analysis. Each trial was recorded for two minutes. We defined the dominant mouse as the mouse with more winning trials and the subordinate mouse as the mouse with fewer.

### Data analysis

#### Behavioral recording

For urine marking tests and home-cage observations, behavioral data were recorded manually by a trainee observer. For the urine marking test, the total number of urine spots was measured across trials and used to assess dominance behavior and sex differences. For the tube test and food competition assay, behavior was recorded using overhead digital video cameras positioned above each apparatus.

#### Urine marking test

The total number of urine spots was measured across trials and used to assess dominance behavior and sex differences. Ties were determined based on whether there was less than a 20% difference or a 5-spot difference in urine.

#### Tube test

Trained observers hand-scored behavior using Behavioral Observation Interactive Software (BORIS). Data from the last day of the test was used in the final analysis, resulting in 36 videos (six cages and six pair combinations per cage). Trained observers manually scored pushing, resisting, active and passive retreat behaviors, and trial duration to compare dominance and sex differences. Scorers identified each behavioral event according to predefined ethograms. Behavioral annotations were exported for subsequent statistical analyses.

#### Food competition

Latency was scored manually from videos recorded on the last day of testing. Shorter trial latencies were used to identify dominance, while longer latencies were used to identify subordinate mice. Once mice were identified as dominant or subordinate, we used the latencies from all their trials to compare social rank during the training and competition tests in both female and male mice. The ranks were calculated as the average latency for each animal and ordered by the number of winning and losing trials. To calculate hierarchy consistency and linearity, data from the last day of competition were analyzed using the Python package HierarchiaPy (v0.2.6). A total of 11 cages (6 female, 5 male) were included in the hierarchy analysis. Winner-loser interactions from food competition trials were combined across testing days to generate a pairwise dominance matrix. For each cage, the Directional Consistency Index (DCI)^60^ and improved Landau’s h (h’) were calculated as measures of hierarchy consistency and hierarchy linearity, respectively. Dominance hierarchy analysis was performed at the cage level, with each cage represented by a single DCI and one Landau’s h (h’) value.

The Directional Consistency Index (DCI) was calculated as:

$$DCI=\frac{\sum \left(H-L\right)}{\sum \left(H+L\right)}$$

where *H* is the greater number of wins and *L* is the lesser number of wins observed within each pairwise interaction. Hierarchy linearity was quantified using the improved Landau’s h statistic (h’) implemented in HierarchiaPy, following Landau^62^ and de Vries^61^.

### Statistical analysis

Statistical analyses included Shapiro–Wilk tests for normality, Welch’s t-test, and one-way and two-way ANOVAs, Pearson’s correlations, and linear regression to visualize best-fit trends in scatterplots.

Behavior schematics were created with BioRender.com

## Supplementary Material

Supplementary Files

This is a list of supplementary files associated with this preprint. Click to download.

- Supplementaryinformation.docx
- Foodcompetitiontraining.mp4
- Foodcompetitiontest.mp4

## Figures and Tables

**Figure 1:**
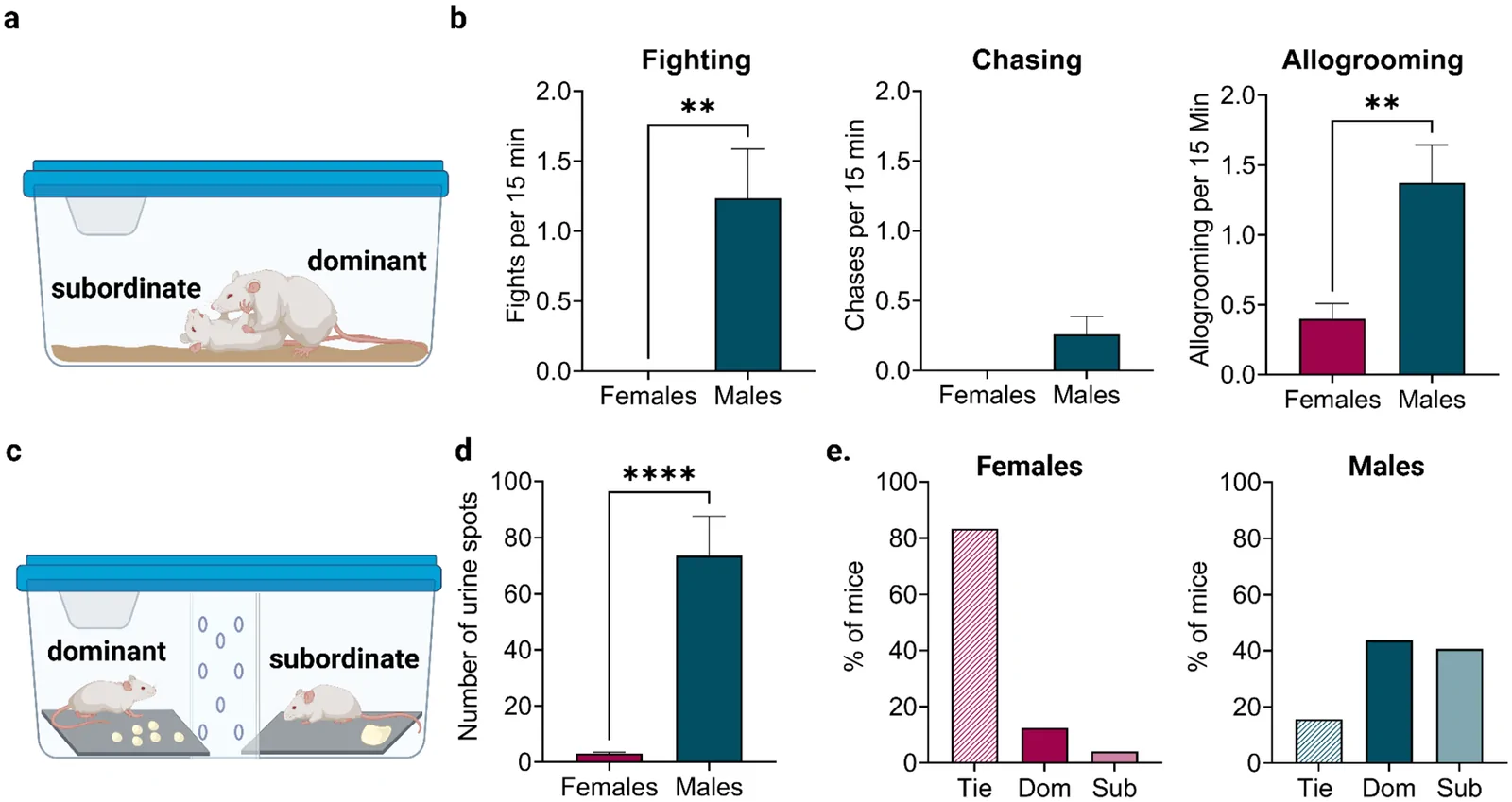
Agonistic behavior and urine behaviors are ineffective in establishing dominance in CD1 female mice. **a,** Schematic of an agonistic behavior assay, with the dominant mouse initiating the behavior and the subordinate mouse receiving the behavior. ***b (left)***, Mean number of fights per 15 minutes for both female (n = 24) and male (n = 29) mice. **b (*middle***), Mean number of chases per 15 minutes for both sexes. **b (*right*)**, Mean number of allogrooming behaviors per 15 minutes for both sexes. **c,** Schematic of urine marking assay. **d,** Number of urine spots between female (n = 24) and male (n = 28) mice. **e,** Percent of mice that tied during the urine marking assay for both males and females. Data are presented as the mean ± SEM. **P< 0.01, ****P<0.0001. Welch’s t-test.

**Figure 2:**
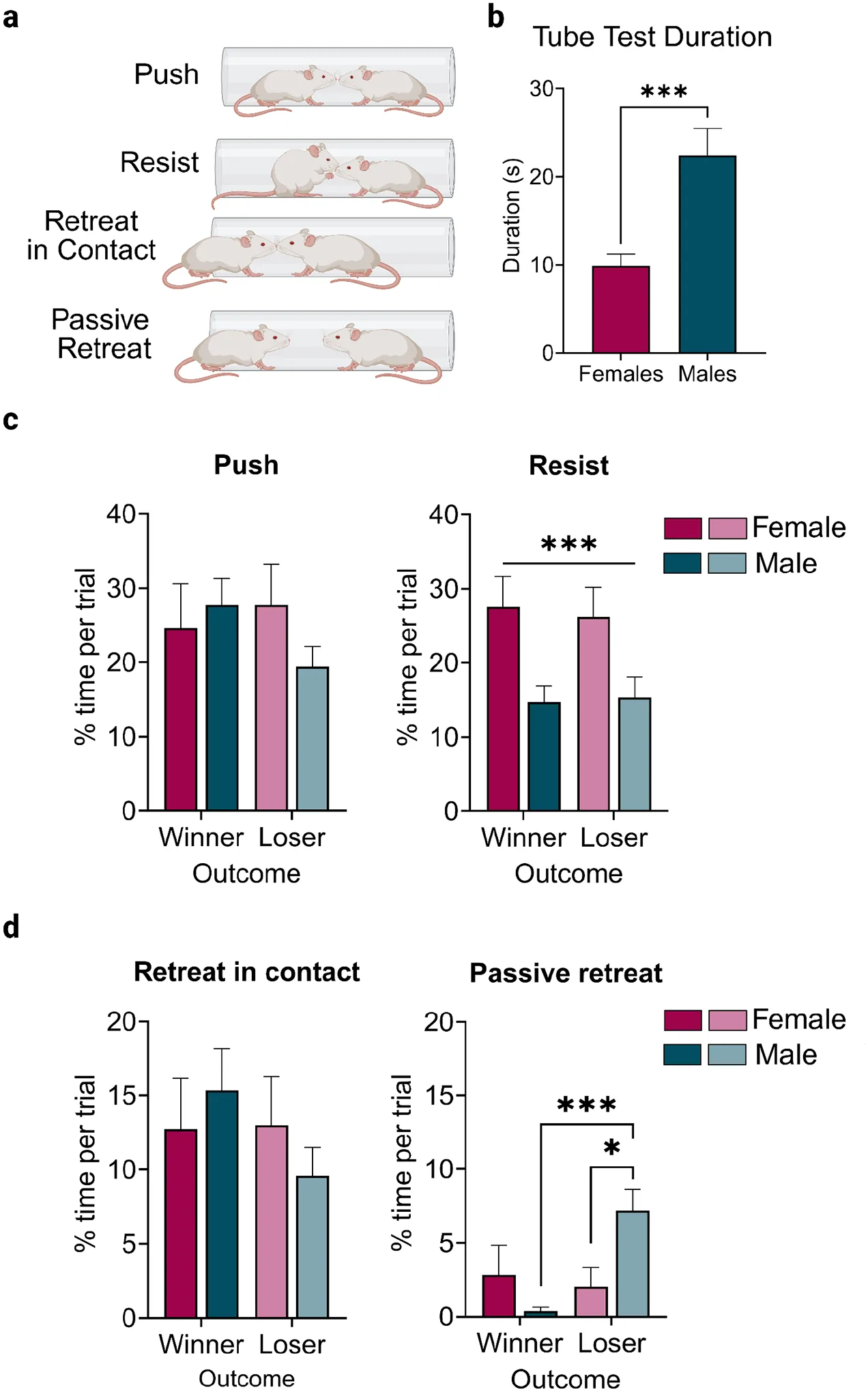
Tube test behavior is insufficient to establish dominance relationships in CD1 females and males. **a,** Schematic of the four tube test behaviors analyzed via the tube test assay. **b,** Mean trial duration for females (n = 24) and males (n = 24) during the tube test assay. **c,** Percentage of tube trial time spent in pushing and resting behavior by winners and losers in females and males. **d,** *Left*, Percentage of tube trial time spent in contact retreat and passive retreat by winners and losers in females and males. Data are presented as the mean ± SEM. **P< 0.01, ***P<0.001. Bonferroni post hoc after two-way ANOVA or Welch’s t-test.

**Figure 3:**
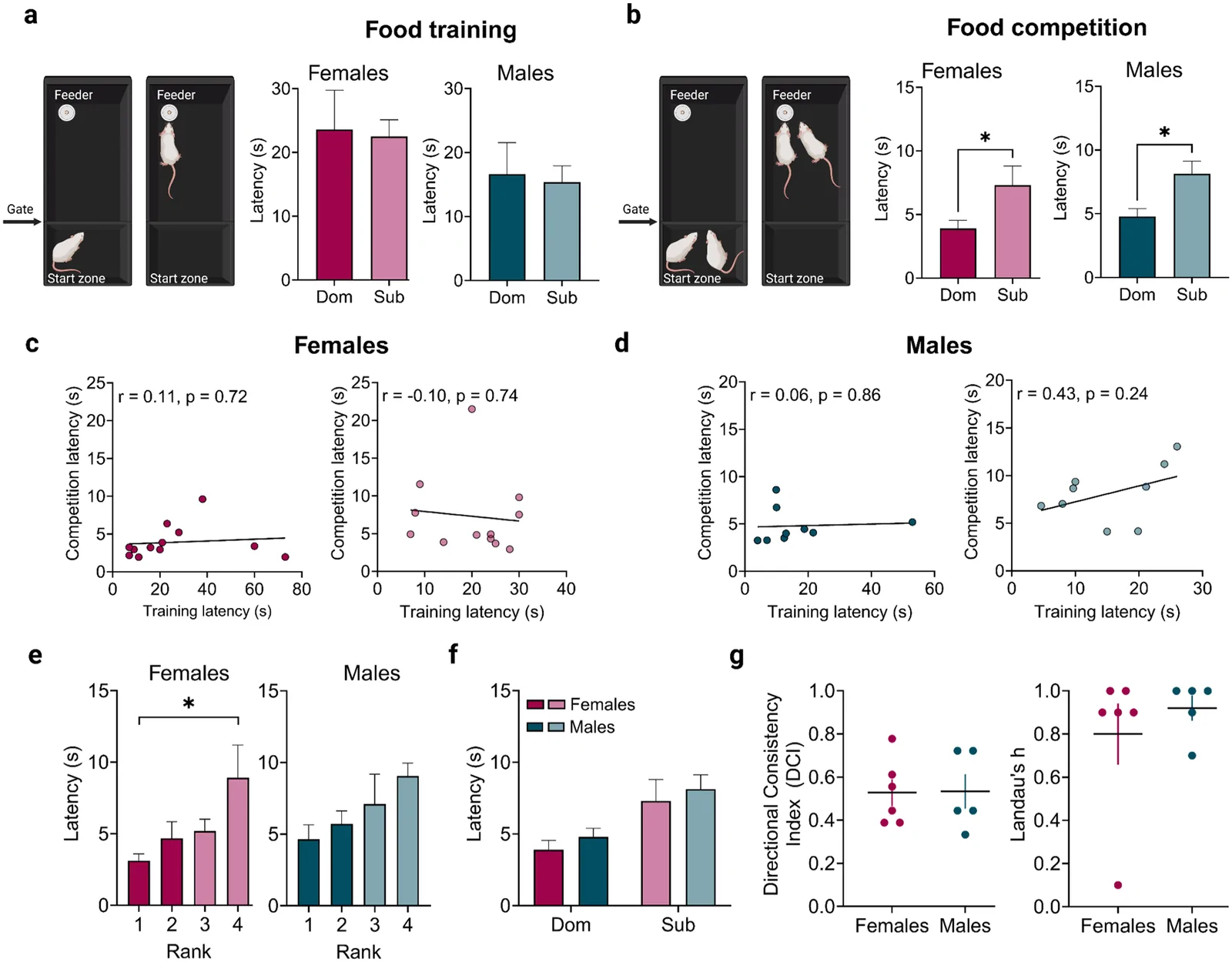
CD1 females and male winners showed shorter latencies during food competition. **a,** *left*, Schematic of the food training assay. *Right*, Latency (s) during the final day of training in dominant (Dom) and subordinate (Sub) female (n = 24) and male (n = 18) mice. **b,** *left*, Schematic of the food competition assay. *Right*, Latency (s) during the food competition test in dominant (Dom) and subordinate (Sub) female and male mice. **c,** Scatterplots showing no significant correlation between training and competition latencies in dominant (left) or subordinate (right) female mice. **d,** Scatterplots showing no significant correlation between training and competition latencies in dominant (left) or subordinate (right) male mice. **e,** Latency across ranks during the competition in females and males. **f,** No sex differences in latency time for dominants or subordinates during competition. **g,** Comparison of hierarchy consistency between female and male groups. *Right*, Comparison of hierarchy linearity between female and male groups. Each point represents an individual social group, and horizontal lines indicate the mean. Data are presented as the mean ± SEM; *P < 0.05. Bonferroni post hoc test after one-way ANOVA, two-way ANOVA, or Welch’s t-test.

## Data Availability

All data needed to evaluate the conclusions in the paper are present in the main text and/or the Supplementary Materials. Additional data related to this paper may be requested from the authors.
